# Effects of overexpression of a bHLH transcription factor on biomass and lipid production in *Nannochloropsis salina*

**DOI:** 10.1186/s13068-015-0386-9

**Published:** 2015-12-01

**Authors:** Nam Kyu Kang, Seungjib Jeon, Sohee Kwon, Hyun Gi Koh, Sung-Eun Shin, Bongsoo Lee, Gang-Guk Choi, Ji-Won Yang, Byeong-ryool Jeong, Yong Keun Chang

**Affiliations:** Department of Chemical and Biomolecular Engineering, KAIST, 291 Daehak-ro, Yuseong-gu, Daejeon, 305-701 Republic of Korea; Advanced Biomass R&D Center, KAIST, 291 Daehak-ro, Yuseong-gu, Daejeon, 305-701 Republic of Korea

**Keywords:** Microalgae, *Nannochloropsis salina*, Transcription factor, Basic helix-loop-helix, Specific growth rate, Fatty acid methyl ester

## Abstract

**Background:**

Microalgae are considered promising alternative energy sources because they consume CO_2_ and accumulate large amounts of lipids that can be used as biofuel. *Nannochloropsis* is a particularly promising microalga due to its high growth rate and lipid content, and the availability of genomic information. Transcription factors (TFs) are global regulators of biological pathways by up- or down-regulation of related genes. Among these, basic helix-loop-helix (bHLH) TFs regulate growth, development, and stress responses in plants and animals, and have been identified in microalgae. We identified two bHLH TFs in the genome of *N. salina* CCMP1776, *NsbHLH1*, and *NsbHLH2*, and characterized functions of *NsbHLH2* that may be involved in growth and nutrient uptake.

**Results:**

We obtained *NsbHLH2* overexpressing transformants of *N. salina* CCMP1776 by particle bombardment and confirmed that these were stable transformants. Quantitative real-time polymerase chain reaction (qRT-PCR) and Western blotting using antibodies against the FLAG tag that was attached at the end of the coding sequence confirmed the expression of the NsbHLH2 protein under various culture conditions. The qRT-PCR results also indicated that the endogenous and transgenic expression of *NsbHLH2* was reduced under stressed conditions. Overexpression of *NsbHLH2* led to increased growth rate in the early growth period, and concomitantly higher nutrient uptake, than wild type (WT). These enhanced growth and nutrient uptake resulted in increased productivities of biomass and FAME. For example, one of the transformants, NsbHLH2 3–6, showed increased biomass productivity by 36 % under the normal condition, and FAME productivity by 33 % under nitrogen limitation condition. Conclusively, the improved growth in the transformants can be associated with the enhanced nutrient uptake. We are currently assessing their potential for scale-up cultivation with positive outcomes.

**Conclusion:**

Overexpression of *NsbHLH2* led to enhanced growth rate and nutrient uptake during the early growth phase, and increased biomass and FAME productivity, especially in the later period under normal and stressed conditions. Based on these results, we postulate that *NsbHLH2* can be employed for the industrial production of biodiesel from *N. salina*.

**Electronic supplementary material:**

The online version of this article (doi:10.1186/s13068-015-0386-9) contains supplementary material, which is available to authorized users.

## Background

There are growing concerns about the limited supply of energy resources and environmental problems from the use of fossil fuels, and this has led to increasing interest in renewable energy sources. Conventional petroleum-based fuels currently account for approximately 90 % of global energy use. High dependency on fossil fuels has caused the exhaustion of oil deposits and global climate change [1]. Among the various alternative energy sources, microalgae have many advantages. For instance, unlike crop-based biofuels and lignocellulosic biomass, microalgae-based biodiesel does not require large-scale arable land for cultivation [2]. Furthermore, microalgae grow rapidly while consuming CO_2_ for photosynthesis, and accumulate large amounts of lipids that can be converted into biodiesel [3].

*Nannochloropsis* spp. are industrial strains of microalgae used for biodiesel production because of their rapid growth rate and high lipid content (up to 47.5 % of biomass) [4]. The efforts to make *Nannochloropsis* sp. economically feasible for biodiesel production include improvements of strains by genetic engineering. Genome and transcriptome data of *Nannochloropsis* spp. have been released recently [4–6] and various genetic modification tools are available, such as homologous recombination and overexpression of target genes [7–9].

Lipid biosynthesis can be enhanced by several ways including nitrogen (N) depletion and stresses in microalgae [10, 11]. These conditions can be employed artificially and/or spontaneously to understand mechanisms behind the accumulation of lipids. In fact, numerous studies have been reported for transcriptomic and proteomic analyses after applying these conditions [10, 12, 13]. N depletion and limitation conditions are most popular for this purpose due to their highest lipid induction, and can be achieved spontaneously in one step [14, 15]. Understanding effects of other environmental stresses are also important, considering that large-scale cultivation of microalgae is destined to outdoor cultivation using PBRs or raceway ponds [16]. We employed two of these stress conditions, N limitation, and osmotic stress, in order to analyze their effects on growth and lipid accumulation in WT, and more importantly in our transformants.

Metabolic engineering has been used to make economically feasible microalgae that have high lipid productivity, mainly through the engineering of individual metabolic enzymes in the lipid biosynthesis pathway [17]. For example, in *Phaeodactylum tricornutum*, the amount of neutral lipid increases by 82 and 60 % following antisense knockdown of putative pyruvate dehydrogenase kinase (PDK) and overexpression of glycerol-3-phosphate dehydrogenase (GPDH), respectively [18, 19]. Although modification of lipid synthesis is regarded as an important strategy, regulation of individual genes related to lipid synthesis, such as acetyl-CoA carboxylase (ACCase), 3-ketoacyl-ACP synthase (KAS), and fatty acid synthases (FAS), rarely leads to significant increase in the lipid content of plants and microalgae [20].

These failures may be in part due to homeostatic or feedback regulation of metabolic pathways. In these regards, there has been recent emphasis on the global regulation of metabolic enzymes, which can be achieved by employing transcription factors (TFs) to alter the expression of multiple enzymes [21]. It has been reported that the lipid content increased following overexpression of the Dof-type TF in *Chlamydomonas* and *Chlorella* [22, 23]. Previous studies of *Nannochloropsis oceanica* IMET1 have examined its TFs and their binding sites through a bioinformatic approach, and this resulted in a predicted transcriptional regulatory network of triacylglycerol (TAG) biosynthesis [24].

TFs with the basic helix-loop-helix (bHLH) motif have important roles in the regulation of stress responses and growth in plants [25]. In particular, MYC2 is a TF with the bHLH motif that regulates the signaling of jasmonate, the plant hormone that provides defense against pests and pathogens and regulation of growth and development [26]. Anthocyanins, which are involved in stress responses in plants, are regulated by MYB and bHLH TFs [25]. Additional research has been reported that cold tolerance is improved in *Arabidopsis* through overexpression of *VabHLH1*, a bHLH TF from the grapevine [27]. In addition, overexpression of *PebHLH35*, a bHLH TF from *Populus euphratica*, enhanced the development and growth of *Arabidopsis*, and improved the tolerance to water-deficit stress [28]. Therefore, these TFs can be employed in improving production of biomass and/or biofuels in microalgae.

In the present study, we identified two bHLH TFs in *N. salina* CCMP1776, named *NsbHLH1* and *NsbHLH2*, and chose *NsbHLH2* that may be involved in growth based on the RNA expression data available for other related *Nannochloropsis* species [29]. We successfully obtained *NsbHLH2* overexpressing transformants of *N. salina* CCMP1776 and cultivated them under normal and stressed conditions including N limitation and osmotic stress. We investigated *NsbHLH2* expression by Western blotting and quantitative real-time PCR (qRT-PCR) and examined the effect of *NsbHLH2* on growth and lipid production.

## Results

### Selection of target bHLHs and identification of transgenic cells

There are 3 isoforms of bHLH TFs in *N. gaditana* HH-1 (Table 1) [13, 29]. Transcriptome data of *N. gaditana* HH-1 indicated that expression of *NgHbHLH1* was greater during the stationary phase than the exponential phase, expression of *NgHbHLH2* was more than 3-times greater during the exponential phase than the stationary phase, and expression of *NgHbHLH3* was very low [29]. Based on the sequence homology, we identified two bHLH TFs in *N. salina*, namely *NsbHLH1* and *NsbHLH2*, but could not find the *NgHbHLH3* homolog (EnergyAlgaeDB: http://www.bioenergychina.org/fg/d.wang_genomes/) (Additional file 1: Figure S1) [4]. We expected that the two pairs of *bHLH* genes were true homologs, because the nuclear and organellar genomes of *N. salina* and *N. gaditana* are very closely related, compared to other *Nannochloropsis* species [4, 30]. We cloned one of the *bHLH* homologs, *NsbHLH2*, expecting that it would be involved in growth particularly during the exponential phase.

**Table 1:** bHLH TFs in *Nannochloropsis* spp

Nannochloropsis Strain	Given name	Genbank or transcript ID	Protein ID	Peptide length	Reference
*N. gaditana* HH-1	*NgHbHLH1*	GAGR01000188		651	[29]
	*NgHbHLH2*	GAGR01001939		599	
	*NgHbHLH3*	GAGR01006326		243	
*N. gaditana* B-31	*NgBbHLH1*	Naga_100055g9	EWM24640	657	[13]
	*NgBbHLH2*	Naga_100009g104	EWM28609	591	Nannochloropsis genome portal^a^
*N. salina* CCMP1776	*NsbHLH1*	KT390183^c^		650	[4]
	*NsbHLH2*	KT390184^c^		605	EnergyAlgaeDB^b^

^a^Nannochloropsis genome portal: http://www.nannochloropsis.org/

^b^EnergyAlgaeDB: http://www.bioenergychina.org/fg/d.wang_genomes/

^c^GenBank accession numbers has been assigned for the CDS sequences

**Fig. 1:**
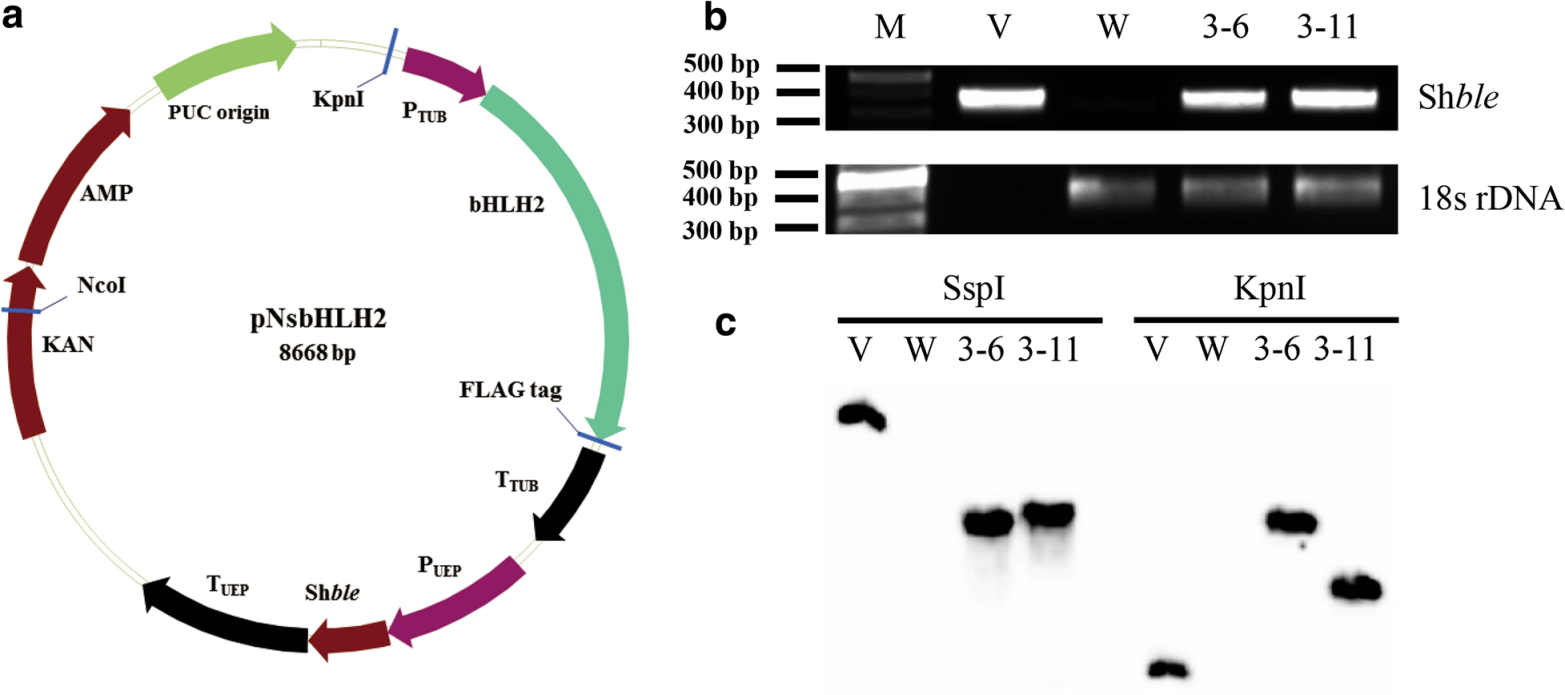
The pNsbHLH2 vector and identification of the plasmid in transformants. **a** Schematic map of the pNsbHLH2 plasmid. **b** PCR detection of the plasmid in two transgenic strains and WT by agarose gel electrophoresis to verify the Sh*ble* PCR product (357 bp) and 18S rDNA (380 bp). **c** Southern blotting of *NsbHLH2* transformants. Genomic DNA was digested by SspI or KpnI, and then hybridized with a 357 bp fragment of the partial Sh*ble* gene. 3–6, 3–11: different *NsbHLH2* transformants; *M* marker; *V* pNsbHLH2 vector; *W* wild type

We constructed a plasmid-harboring *NsbHLH2* that was controlled by the endogenous *TUBULIN* (*TUB*) promoter and named the vector pNsbHLH2 (Fig. 1a). This vector also contained a selectable marker Sh*ble* conferring resistance to Zeocin, an antibiotics resembling bleomycin. pNsbHLH2 was transferred into *N. salina* cells by particle bombardment, and the transformed cells were plated on F2 N agar medium containing 2.5 μg/mL Zeocin. After 3–4 weeks, we selected Zeocin-resistant colonies. We performed the Western blot to detect expression of the FLAG tag that was attached at the C-terminus of the NsbHLH2 protein at the expected size of 65 kD (Additional file 2: Figure S2). These strains were screened for growth and lipid contents based on optical density and Nile red fluorescence, respectively, and we selected NsbHLH2 3–6 and 3–11 transformants for further analyses (Additional file 3: Figure S3, Additional file 6: Text S1).

In order to check the presence of the plasmid DNA in transformants, we conducted genomic DNA PCR (Fig. 1b). PCR was performed using the S1 and S2 primers to detect Sh*ble* and the SR6 and SR9 primers to detect 18S rDNA (Additional file 5: Table S1). The Sh*ble* gene was detected only in the transformants 3–6 and 3–11, but not in WT, while 18S rDNA was detectable in all samples except for the vector control, indicating successful transformation of *N. salina*.

We confirmed integration of the transgene into genomic DNA by performing the Southern blot (Fig. 1c). Using the Sh*ble* probe, we found that the transgenic Sh*ble* gene was clearly present in both transformants, but not in WT. Each transformant had a single band with different sizes, indicating that the transformants contained a single copy of Sh*ble* integrated at different locations of the genome. Taken together, these results indicate that the pNsbHLH2 plasmid was integrated into the genomic DNA of the two transformants and was stably and correctly expressed by the cells.

### Molecular analysis of NsbHLH2 transformants

**Fig. 2:**
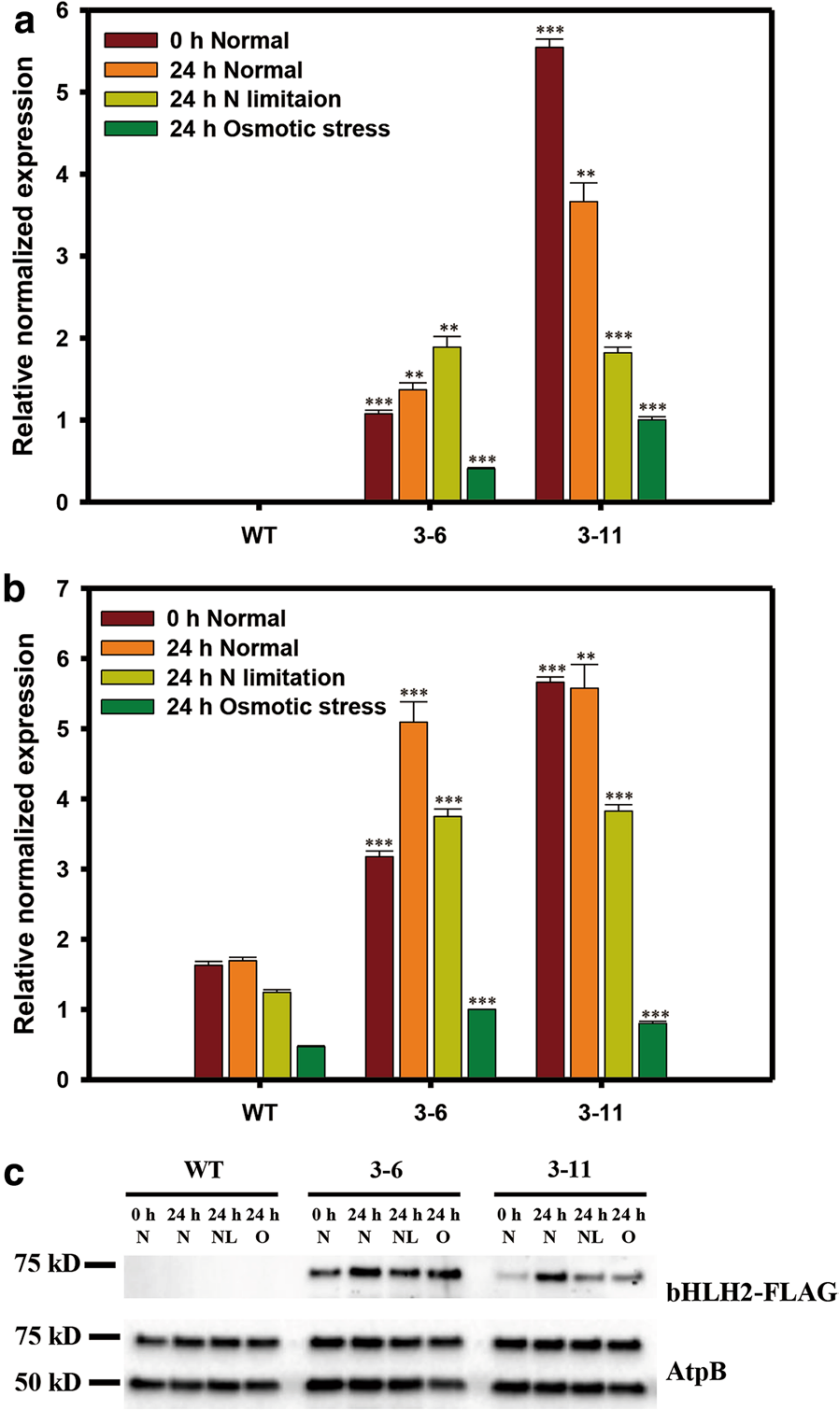
Expression of *NsbHLH2* in transformants under different culture conditions. **a** qRT-PCR of transgenic NsbHLH2 mRNA by using qbH1 and qbH2 primers, where qbH1 is located in the vector. **b** qRT-PCR of transgenic and endogenous NsbHLH2 mRNA by using qbH3 and qbH4 primers. **c** Western blotting of FLAG-tagged NsbHLH2. The expected size of FLAG-tagged NsbHLH2 was 65 kD. AtpB [expected sizes of 72.6 kD (F-type H-ATPase *ß* subunit) and 53.13 kD (CF_1_ *ß* subunit of ATP synthase)] was used as a loading control. Accession number of CF_1_ *ß* subunit of ATP synthase from *N. salina* CCMP537 is YP_008519835; accession number of F-type H-ATPase *ß* subunit from *N. gaditana* B-31 is EWM25142. Homologs of these proteins were present in *N. salina* CCMP1776, and appeared to be good loading controls with constant expression level under different culture conditions. *WT* wild type; *N* normal conditions; *NL* nitrogen limitation; *O* osmotic stress. The data points represent the average of samples and *error bars* indicate standard error (*n* = 3). Significant differences, as determined by Student’s *t* test, are indicated by asterisks (*P < 0.05, **P < 0.01, ***P < 0.001)

We also measured the expression of endogenous and transgenic *NsbHLH2* under different culture conditions using qRT-PCR and Western blotting (Fig. 2). For these experiments, samples were collected at 0 h under normal conditions and at 24 h after the change in culture conditions (normal, N limitation, and osmotic stress condition). First, we examined the transcription of transgenic *NsbHLH2* using the forward primer in the untranslated region of the plasmid (qbH1) in combination with the reverse primer located in the coding sequence of *NsbHLH2* (qbH2) (Additional file 5: Table S1). These primers amplified correct products only in the transformants, and qRT-PCR revealed variable expression of the transgenic *NsbHLH2* (Fig. 2a). Although the constitutive *TUB* promoter was used to express *NsbHLH2*, expression depended on the culture conditions. In particular, there was significantly reduced expression of *NsbHLH2* under osmotic stress. We also used Western blot to show expression of FLAG-tagged NsbHLH2 under the three different culture conditions. Consistent with the qRT-PCR results, the FLAG-tagged proteins were found only in the transformants (Fig. 2c). However, expression pattern was not consistent with that of RNAs. Expression of the NsbHLH2 protein was rather constant and high in transformant 3–6, while that of 3–11 was low except for the 24 h sample under the normal condition. These discrepancies suggest that accumulation of transgenic NsbHLH2 protein mainly depends on the stability of the protein rather than on the amount of RNAs.

Additionally, we assessed expression of total NsbHLH2 RNAs including endogenous and transgenic copies of *NsbHLH2* (Fig. 2b). Overall, total quantities of NsbHLH2 RNAs in the transformants were higher than WT, indicating additive accumulation of NsbHLH2 RNA. Interestingly, we found a tendency that the RNA level decreased when cells were stressed: N limitation caused reduction in the amount of NsbHLH2 RNAs, and osmotic stress resulted in dramatic reduction in the RNA level. Interpretation of these results, together with functional characterization of NsbHLH2 transformants, will be further discussed.

### Growth of NsbHLH2 transformants under different culture conditions

**Fig. 3:**
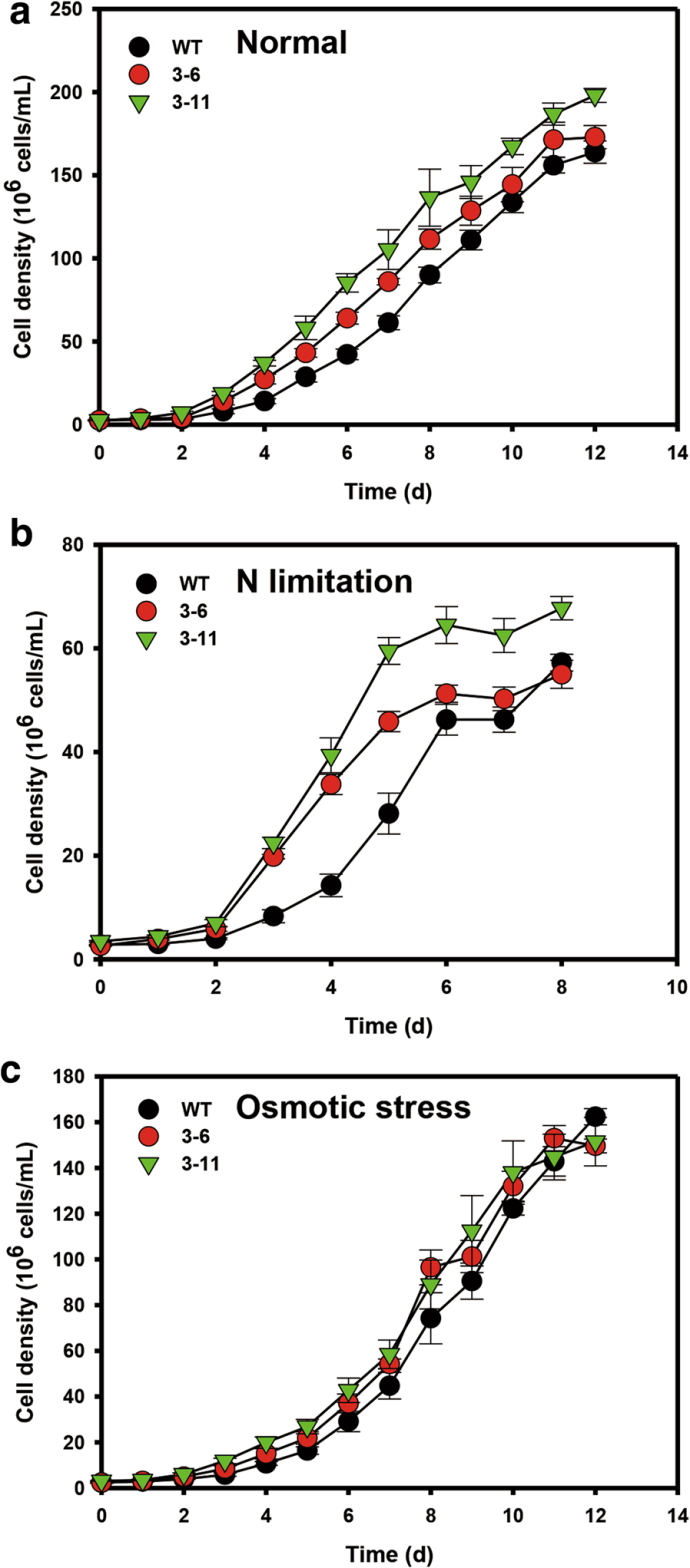
Growth analyses of *NsbHLH2* transformants under various culture conditions. Growth curve based on cell density under normal condition (**a**), N limitation (**b**), and osmotic stress condition (**c**). Cells were cultivated at 25 °C, 120 rpm, 120 µmol photons/m^2^/s of fluorescent light, and 0.5 vvm of 2 % CO_2_. Data points represent means and standard errors (*n* = 4)

We analyzed the growth of WT and *NsbHLH2* transformants under three different culture conditions: normal F2 N medium, F2 N medium with N limitation, and F2 N medium with osmotic stress. N limitation was administered by reducing nitrate concentration to 0.88 mM in the F2 N medium, and osmotic stress by adding sea salt to 50 g/L. Overall transformants showed better growth compared to WT in all three conditions (Fig. 3). It should be noted that experiments with N limitation were ended earlier due to lack of growth. While observing the growth pattern, we noticed that the growth of transformants was faster at the initial period of the growth phase, which remained high at the later phases of growth. Therefore, we divided the culture period into three: the initial period from days 0 to 4, the middle period for days 4–8, and the late period for days 8–12. Specific growth rates for these periods were estimated as summarized in Table 2. As expected from the growth pattern, specific growth rate of transformants at the initial period was significantly higher than the rest of the period. In particular, under N limitation, the specific growth rates of the transformants were 60 % higher (for transformant 3–6) and 52 % higher (for 3–11) than WT. However, specific growth rates of the transformants were similar or even lower than WT at later periods. In an effort to resolve this poor performance of transformants at the later stages of cultivation, we attempted a preliminary fed-batch experiment where nutrients were supplemented at intervals to one of the transformants NsbHLH2 3–11. Compared to WT, it showed remarkable improvements in the growth rate particularly during the later stage of 14-day cultivation. The cell number of NsbHLH2 3–11 was 70 % higher than that of WT (Additional file 4: Figure S4, Additional file 6: Text S1).

**Table 2:** Specific growth rate, biomass productivity, and FAME content of WT and two *NsbHLH2* transformants

Culture condition	Strain	Specific growth rate (day^−1^)^a^			Biomass productivity (mg/L/day)		FAME content (%)	
		Day 0–4^b^	Day 4–8^c^	Day 8–12^d^	8 day	12 day	8 day	12 day
Normal	WT	0.43 ± 0.04	0.47 ± 0.03	0.15 ± 0.01	89.6 ± 8.8	168.8 ± 10.4	15.6 ± 0.8	21.7 ± 0.9
	3–6	0.59 ± 0.03**	0.35 ± 0.04	0.11 ± 0.02	121.9 ± 11.5*	176.0 ± 9.7	16.5 ± 0.7	24.5 ± 1.2
	3–11	0.67 ± 0.03**	0.32 ± 0.03	0.10 ± 0.03	133.3 ± 23.0	170.8 ± 9.9	16.5 ± 1.1	24.1 ± 1.6
N limitation	WT	0.40 ± 0.04	0.36 ± 0.36		78.1 ± 2.4		42.3 ± 1.4	
	3–6	0.64 ± 0.02**	0.12 ± 0.01		96.3 ± 5.4*		46.0 ± 3.3	
	3–11	0.60 ± 0.03**	0.14 ± 0.02		93.7 ± 6.3*		41.5 ± 1.2	
Osmotic stress	WT	0.38 ± 0.04	0.48 ± 0.03	0.21 ± 0.04	102.1 ± 12.3	168.7 ± 12.1	17.2 ± 0.9	30.1 ± 3.0
	3–6	0.46 ± 0.04	0.47 ± 0.05	0.11 ± 0.02	126.0 ± 15.9	180.1 ± 4.9	17.8 ± 1.2	32.5 ± 3.8
	3–11	0.46 ± 0.02	0.38 ± 0.01	0.14 ± 0.02	131.2 ± 19.6	153.1 ± 4.6	17.2 ± 0.4	29.9 ± 4.7

The data points represent the average of samples and error bars indicate standard error (*n* = 4). Significant differences, as determined by Student’s *t* test, are indicated by asterisks (* *P* < 0.05, ** *P* < 0.01, *** *P* < 0.001)

^a^Specific growth rate was calculated as (*μ*/day) = ln(*X*_2_/*X*_1_)/(*t*_2_ − *t*_1_); where *X*_1_ and *X*_2_ are the initial and final biomass and *t*_1_ and *t*_2_ are the initial and final times

^b^Specific growth rate based on cell density on day 0 and day 4

^c^Specific growth rate based on cell density on day 4 and day 8

^d^Specific growth rate based on cell density on day 8 and day 12

### Biomass and FAME analyses of NsbHLH2 transformants

**Fig. 4:**
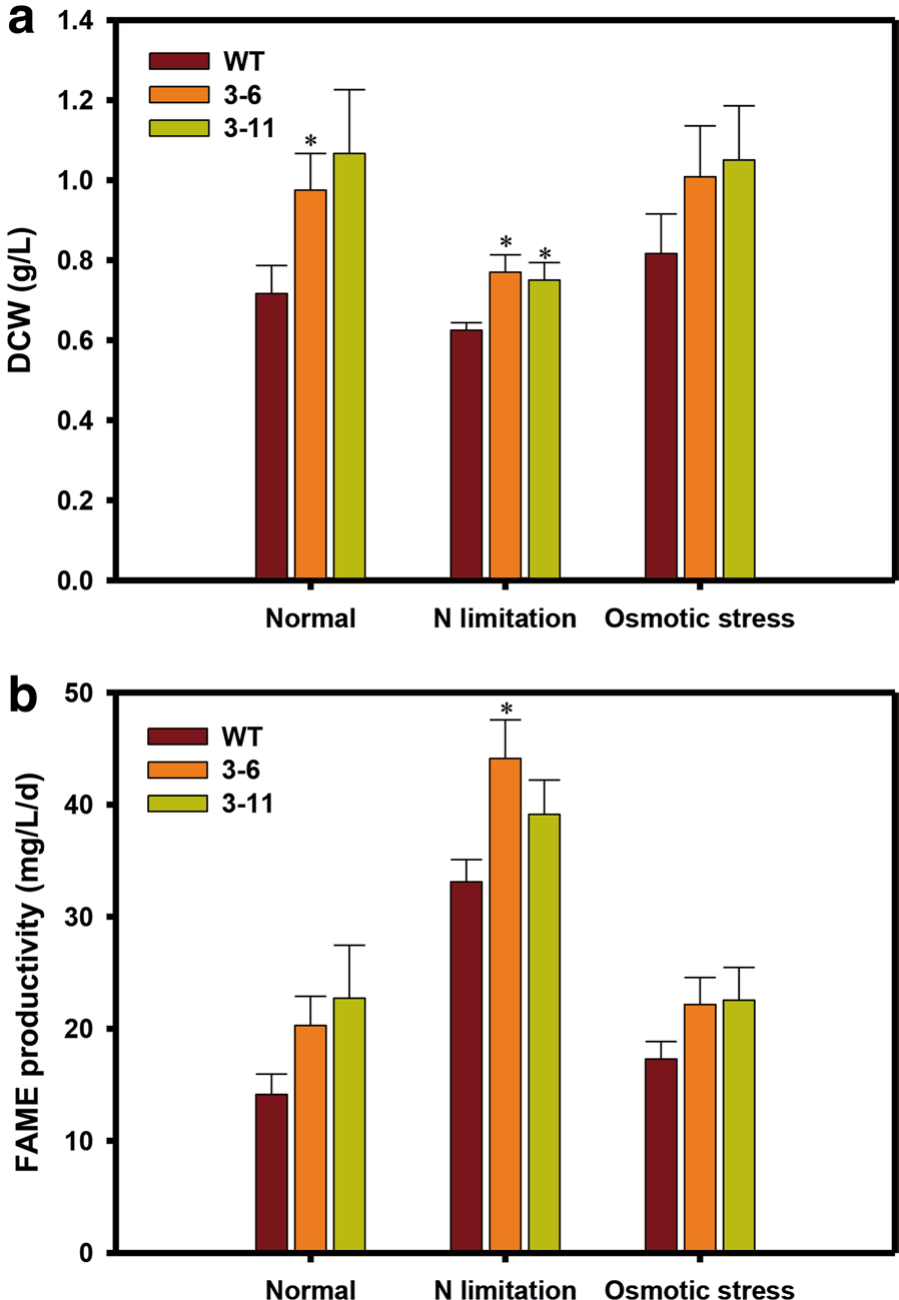
DCW and FAME productivity of *NsbHLH2* transformants under various culture conditions. The biomass for DCW (**a**) and FAME productivity (**b**) was obtained at day 8. Cells were cultivated at 25 °C, 120 rpm, 120 µmol photons/m^2^/s of fluorescent light, and 0.5 vvm of 2 % CO_2_. The data points represent the average of samples and *error bars* indicate standard error (*n* = 4). Significant differences, as determined by Student’s *t* test, are indicated by *asterisks* (*P < 0.05, **P < 0.01, ***P < 0.001)

The high growth rate at early growth phase of the transformants was also associated with a greater dry cell weight (DCW) and biomass productivity at day 8 (Fig. 4; Table 2). Under the normal condition, DCW of the NsbHLH2 3–6 transformant at 8th day was increased by 36 % (Fig. 4a). Under N limitation and osmotic stress, the DCW of the transformants at 8th day was more than 20 % greater than WT (Fig. 4a). However, biomass productivity of all strains was similar at day 12 under normal conditions and under osmotic stress (Table 2).

We measured the FAME content and productivity on days 8 and 12 to evaluate the potential use of *NsbHLH2* transformants for biodiesel production (Fig. 4b). The overall FAME contents of the two transformants were similar with one another, and with WT, under normal culture conditions (Table 2). However, since *NsbHLH2* transformants accumulated more biomass, their FAME productivity was significantly higher than WT. Especially at day 8 under N limitation, FAME productivity of NsbHLH2 3–6 at day 8 was 44.12 mg/L/day, 33.2 % greater than WT (Fig. 4b). Under normal and osmotic stress condition, FAME productivity rates of NsbHLH2 transformants were also 43 and 28 % greater than WT under normal and osmotic stress conditions, respectively, even though there was no statistical significance (Fig. 4b). Less increase of FAME productivity was observed on day 12 under the same condition (Table 2).

**Fig. 5:**
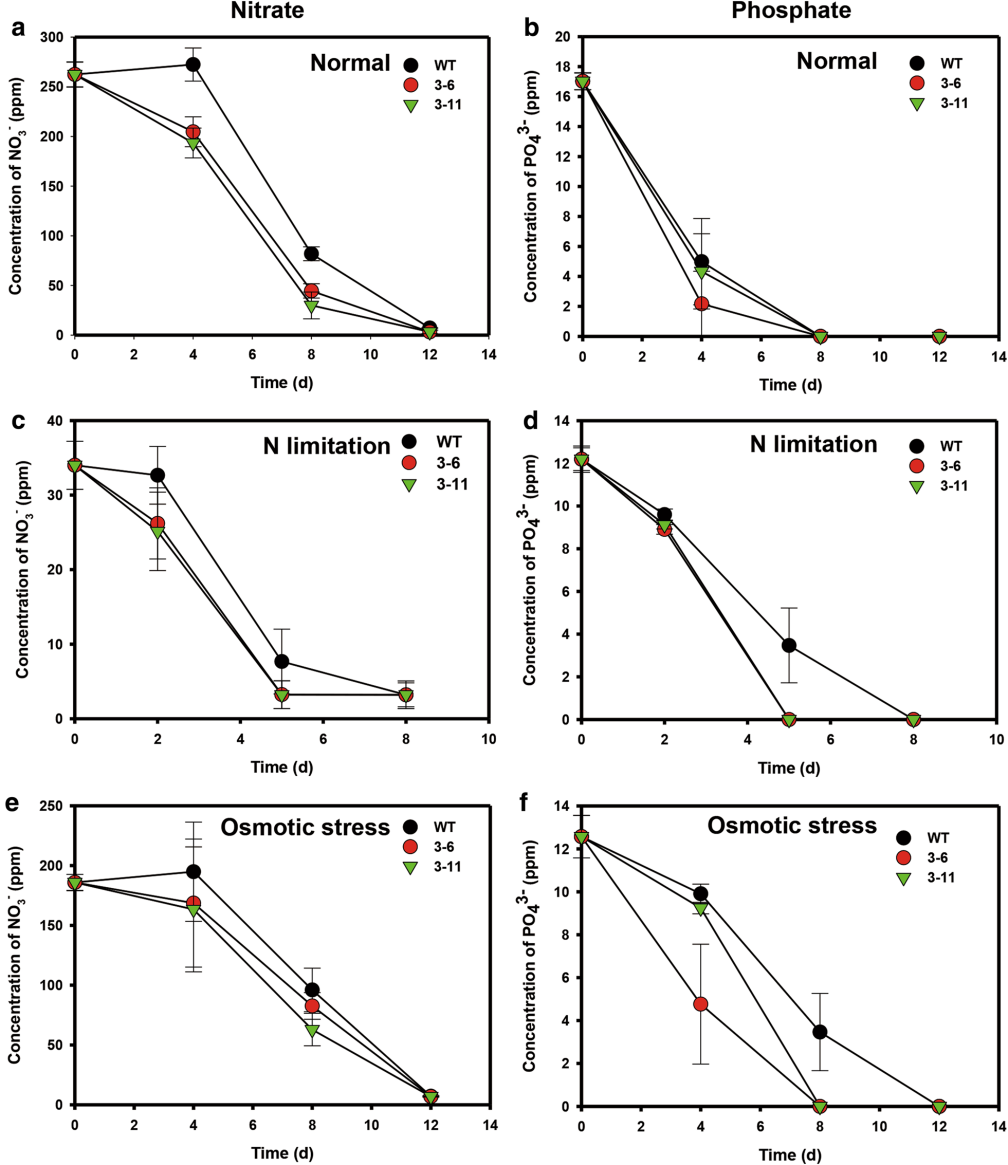
Nutrient consumption during growth of *NsbHLH2* transformants. **a** Nutrient uptake was estimated by measuring concentrations of nitrate (**a**, **c**, **e**) and phosphate (**b**, **d**, **f**) in the culture media under normal (**a**, **b**), N limitation (**c**, **d**), and osmotic stress conditions (**e**, **f**). Cells were cultivated at 25 °C, 120 rpm, 120 µmol photons/m^2^/s of fluorescent light, and 0.5 vvm of 2 % CO_2_. Data points represent means and standard errors (*n* = 4)

In order to investigate the mechanism behind increased accumulation of biomass and FAME productivity, we analyzed nutrient uptake in the *NsbHLH2* transformants (Fig. 5). The *NsbHLH2* transformants consumed nitrate and phosphate more rapidly under all culture conditions, in parallel with their rapid growth during the early growth phase (Fig. 3). However, as most of the nutrients were consumed by day 8, the specific growth rates, FAME productivity, and DCWs of the transformants became similar with those of WT (Fig. 4; Table 2).

## Discussion

*Nannochloropsis* has been used for commercial purposes, and now is considered a model microalga for biofuel production due to the high biomass productivity and lipid contents with the possibility of large-scale cultivation [31]. In addition, there is growing interests in the genetic manipulation of *Nannochloropsis* for the production of commercial biodiesel. Even though genome and genetic tools of *Nannochloropsis* are available, no studies have been reported regarding genetic engineering of *Nannochloropsis* for improved biodiesel production [4–6], except that a report was published online regarding enhancement of polyunsaturated fatty acids by overexpression of a desaturase while this manuscript was being prepared [9]. In general, genetic engineering for improved lipid productivity in other microalgae has mainly focused on individual metabolic genes, such as ACCase, KAS, and diacylglycerol acyltransferase (DGAT) [32]. However, it may be a better approach to regulate overall metabolism within the cell, rather than focusing on a single enzyme or pathway. Thus, manipulation of TFs that regulate numerous enzymes may be an attractive idea for the genetic improvement of microalgae [21]. Furthermore, TFs are well known as stress regulators, so overexpression of these TFs may be effective because microalgae generally accumulate large amounts of lipids under stressful conditions [29]. The present study is the first to demonstrate that overexpression of a bHLH TF in *N. salina* increases the biomass and production of FAMEs.

In general, microalgae undergo cell division and growth during the exponential phase, and accumulate lipids during the subsequent stationary phase [33]. Therefore, gene clusters that regulate cell division and lipid accumulation are commonly separate. Previous research on *Chlamydomonas reinhardtii* reported that the gene cluster associated with lipid metabolism had high expression during the lipid accumulation phase, and the gene cluster associated with photosynthesis and basic metabolic pathways had low expression during the stationary phase [34]. In *N. oceanica*, genes associated with photosynthesis, ribosomal function, and DNA replication were down-regulated in response to N starvation [10]. In *N. gaditana*, genes responsible for energy metabolism (photosynthesis and respiration) were also down-regulated under N starvation [13]. Therefore, genes related to energy metabolism, growth, and cell division are generally highly expressed under favorable growth conditions, but have lower expression under stressful conditions and during the stationary phase. On the other hand, genes responsible for lipid metabolism generally have low expression under normal conditions, and elevated expression under stressful conditions.

We overexpressed *NsbHLH2* using an endogenous *TUB* promoter to produce transformants that had increased growth and biomass productivity. Even though the *TUB* promoter is considered to be constitutive, expression of the NsbHLH2 RNA changed under different culture conditions (Fig. 2a, c). In particular, *NsbHLH2* expression of transformants decreased dramatically under the osmotic stress, based on our qRT-PCR results (Fig. 2a). In agreement, previous studies reported that β-tubulin of various organisms, including unicellular algae such as *Symbiodinium*, varied following changes in culture conditions [35–37]. In *Nannochloropsis*, *TUB* expression also depends on culture conditions [38]. Even though NsbHLH2 expression was affected by culture conditions, the total level of NsbHLH2 in the transformants was greater than in WT under all tested culture conditions (Fig. 2b). The degree of increase of the NsbHLH2 RNA was higher under the normal and N limitation conditions, compared to the osmotic stress. Consistently, we observed faster growth of *NsbHLH2* transformants under normal and N limitation conditions, and to a less degree under the osmotic stress. The faster growth of transformants was more prominent when we analyzed specific growth rates in the early growth phase during the first four days. This early enhancement of growth appears to remain during the course of experiment, where the growth of transformants remain higher than WT (Fig. 2a). The high growth rate during the early growth phase also positively affected dry cell weight (DCW) and biomass productivity during the later period (Fig. 4a; Table 2). However, the difference between the transformants and WT decreased over time because total *NsbHLH2* expression was low under unfavorable culture conditions and during the stationary phase (Fig. 2) [29].

TFs with bHLH motifs are well known as stress regulator in plants, so we investigated the phenotypes of *NsbHLH2* transformants under stressful conditions (N limitation and osmotic stress). The effect of *NsbHLH2* overexpression on growth was confirmed under N limitation. In particular, the specific growth rate increased during first 4 days under N limitation and the cell density of transformants was 2.3fold greater than WT at day 4, and biomass productivity was higher than WT at day 8 (Fig. 4a; Table 2). This may suggest that the endogenous functions of *NsbHLH2* include growth under normal and N limitation conditions. However, under osmotic stress, effects of *NsbHLH2* overexpression was not pronounced as compared to those of normal and N limitation conditions (Table 2; Fig. 3), and this is consistent with the results of qRT-PCR that expression of both endogenous and transgenic *NsbHLH2* was greatly reduced. Nevertheless, transformants showed significantly increased expression of total NsbHLH2 RNA, and we observed increased growth rate and biomass productivity under osmotic stress.

Our nutrient consumption data are consistent with the faster growth of transformants during the early stages of culture (Fig. 5). Compared to WT, transformants consumed nitrate and phosphate more rapidly under all culture conditions, probably due to their fast growth during first 4 days. By consuming these nutrients rapidly, the transformants had greater DCW at day 8 than WT. However, when all nutrients were consumed completely (day12), the specific growth rate and DCW of transformants and WT were similar. Therefore, overexpression of *NsbHLH2* enhances growth because it leads to rapid consumption of nutrients, especially during the early growth phase, under all tested culture conditions. This interpretation is supported by our preliminary fed-batch experiment that showed sustained increase in overall growth rate in NsbHLH2 3–11 compared to WT. This concept will be conveyed to our further studies of the scale-up cultivation for improved biomass production.

Overexpression of *NsbHLH2* in *N. salina* also has advantages for the efficient production of biodiesel. The transformants had high FAME productivity under all tested culture conditions, especially at day 8 (Fig. 4b), in parallel with their high DCW. In general, FAME productivity is a function of DCW and FAME content. Because the NsbHLH2 TF seemed to have little effect on FAME content, high biomass productivity due to fast growth rate for first 4 days can be attributed to high FAME productivity on day 8 (Table 2; Fig. 4b). The specific growth rate of WT increased after the early growth phase, and there was only a small difference in the biomass of the transformants and WT on day 12. As a result, total FAME productivity of the transformants on day 12 was similar to that of WT. Nevertheless, FAME productivity of the transformants was more than 13 % greater than WT on day12 (except for transformant 3–11 under conditions of osmotic stress).

In this study, we confirmed that the NsbHLH2 TF had a role in cell growth and division, particularly during the early growth phase. Moreover, transformants that overexpressed *NsbHLH2* had a faster growth rate and greater biomass productivity during the early part of the growth curve. In addition, because of their fast growth rate, these transformants also produced more FAMEs during the early part of the growth curve. Although further studies of mechanistic relationship between the overexpression of NsbHLH2 and increased productivity of biomass and lipid are needed, our results establish the feasibility of increasing biodiesel production by the genetic engineering of a TF in *N. salina*.

## Conclusion

The present study demonstrated that overexpression of the NsbHLH2 TF in *N. salina* increased cell growth and production of FAMEs. Our qRT-PCR and Western blotting experiments confirmed that the function of NsbHLH2 TF was mainly related to growth and cell division. Expression of NsbHLH2 TF was elevated under favorable culture conditions, while decreased under stress conditions, such as N limitation and osmotic stress. However, the higher expression of *NsbHLH2* in the transformants was associated with a fast specific growth rate during the early growth phase (first 4 days), resulting in an increased DCW and FAME productivity, especially at day 8. These findings suggest that overexpression of the NsbHLH2 TF in *N. salina* and probably other industrial strains of microalgae has potential use for biodiesel production under different culture conditions.

## Methods

### Microalgal strain and culture conditions

*Nannochloropsis salina* CCMP1776 from National Center for Marine Algae and Microbiota (formerly Culture Collection of Marine Phytoplankton, CCMP) was maintained in modified F2 N medium [39], which consisted of 15 g/L sea salt (Sigma-Aldrich, USA), 10 mM Tris–HCl (pH 7.6), 427.5 mg/L NaNO_3_, 30 mg/L NaH_2_PO_4_∙2H_2_O, 5 mL/L trace metal mixture (4.36 g/L Na_2_ EDTA∙2H_2_O, 3.15 g/L FeCl_3_∙6H_2_O, 10 mg/L CoCl_2_∙ 6H_2_O, 22 mg/L ZnSO4∙7H_2_O, 180 mg/L MnCl_2_∙4H_2_O, 9.8 mg/L CuSO4∙5H_2_O, 6.3 mg/L Na_2_MoO_4_∙2H_2_O), and 2.5 mL/L vitamin stock (1 mg/L vitamin B_12_, 1 mg/L Biotin, 200 mg/L thiamine∙HCl) [40]. Cells were cultivated in 250 mL Erlenmeyer baffled flasks with 200 mL working volumes at 25 °C with agitation (120 rpm) under the fluorescent light (120 µmol photons/m^2^/s). Air with 2 % CO_2_ was supplied to the broth culture at 0.5 vvm (volume gas per volume medium per minute).

### Growth and nutrient analysis of broth culture

Wild-type and *NsbHLH2* transformants were cultivated under normal conditions (F2 N medium), nitrogen limitation (F2 N medium with NaNO_3_ concentration decreased to 75 mg/L), and osmotic stress (F2 N medium with sea salt concentration increased to 50 g/L). The cultivation conditions were as follows: 25 °C, 120 rpm, 120 µmol photons/m^2^/s fluorescent light, and 0.5 vvm of air containing 2 % CO_2_. Cell growth was determined by measuring cell density (in cells/mL) and dry cell weight (DCW). Cell density was determined by a hemocytometer, and DCW was estimated by filtering cells with the GF/C filter paper (Whatman, USA), washing with deionized water, drying at 105 °C overnight, and weighing on a fine scale. The specific growth rate was calculated as

1 $${\text{Specific growth rate }}\left( {\mu / {\text{day}}} \right) {\text{ = ln}}\left( {X_{ 2} /X_{ 1} } \right) / (t_{ 2} { - }t_{ 1} )$$

where *X*_1_ and *X*2 are the initial and final cell density and *t*_1_ and *t*_2_ are the initial and final times. The concentrations of nitrate (NO_3_^−^) and phosphate (PO_4_^3−^) in the broth were determined by ion chromatography (881 compact IC pro, Metrohm, Swiss) with a Metrosep A Supp5 150 column for anions.

### Vector construction

After cell harvest at the mid-exponential phase, 200 mg of wet biomass was used to obtain total RNA. The total RNA was extracted using the RNeasy Plant mini kit (Qiagen, USA) and DNA was removed using the DNA-free™ DNase kit (AMBION, USA) following manufacturer’s instructions. The cDNA was synthesized using Superscript™ III Reverse Transcriptase (Invitrogen, USA) and an oligo (dT)_20_ primer (Invitrogen, USA). The coding sequence of *NsbHLH2* was amplified from *N. salina* cDNA using primers bH1 and bH2 (Additional file 5: Table S1). These primer sequences were determined by comparison of sequences from strains of *N. gaditana* and *N. salina* (EnergyAlgaeDB: http://www.bioenergychina.org/fg/d.wang_genomes/) [4]. The backbone of pNsbHLH2 was amplified using primers bH3 and bH4. The plasmid pNsbHLH2 (Fig. 1) harbors the endogenous TUB promoter (the constitutive promoter of β-tubulin-coding gene) and terminator for expression of the *NsbHLH2* gene. The selection marker Sh*ble* gene that confers resistance to Zeocin (Invitrogen, USA) was expressed by the constitutive *UEP* (encoding the ubiquitin extension protein) promoter and terminator. The plasmid was constructed by the Gibson assembly technique [41].

### Particle bombardment

For particle bombardment transformation, the pNsbHLH2 plasmid was linearized by NcoI. This linearized plasmid was purified, concentrated to 1 μg/μL by ethanol precipitation, and coated onto 0.6 μm microcarrier gold particles (Bio-Rad, USA). For bombardment, 3 mg of gold particles in 50 μL of 50 % glycerol was vigorously mixed with 6 μL of purified DNA, 50 μL of 2.5 M CaCl_2_, and 20 μL of 0.1 M spermidine. The gold DNA-coated particles were washed by 70 % ethanol and resuspended in 60 μL of 100 % ethanol. For each bombardment, 12 μL of DNA in 100 % ethanol was used.

*N. salina* in the mid-exponential phase (OD_680nm_ = 6) was concentrated, and 10^8^ cells on cellulose acetate membrane filters were placed on the F2 N agar medium. Particle bombardment was performed by a GDS-80, low-pressure gene delivery system (Wealtec, USA) under 700 psi helium at a 3-cm target distance. After bombardment, cells on cellulose acetate membrane filters were transferred to F2 N broth medium and maintained at 25 °C under 5 µmol photons/m^2^/s of fluorescent light for 1 day, and then plated on selective F2 N agar medium containing 2.5 µg/mL of Zeocin. After 3 weeks, colonies were selected for further analysis.

### Molecular analysis of transformants by genomic PCR and Southern blotting

For genomic PCR, crude DNA of the WT and transformants were extracted by use of Instagene Matrix (Bio-Rad, USA) following modified protocols originally developed by Wan et al. [42]. After harvesting and washing *Nannochloropsis* cells, 200 µL of Instagene Matrix was added, the mixtures were incubated at 56 °C for 20 min, and then at 100 °C for 8 min. After centrifugation, the supernatant was used as a template for genomic PCR. The Sh*ble* gene was detected by S1 (forward) and S2 (reverse) primers, and 18S rDNA was detected by SR6 (forward) and SR9 (reverse) primers (Additional file 5: Table S1) [43]. Ex taq polymerase (Takara, Japan) was used for PCR amplification under the following conditions: 95 °C for 5 min, 30 cycles of 95 °C for 1 min, 60 °C for 1 min, 72 °C for 1 min, and then 72 °C for 10 min. The sizes of the Sh*ble* and 18S rDNA PCR products were 357 bp and 380 bp, respectively.

For Southern blotting, genomic DNAs were isolated following Jeong et al., and probes were prepared using the DIG-High Prime DNA Labeling and Detection Starter Kit II (Roche, Germany) [44]. DNA templates for the Sh*ble* and NsbHLH2 probes were produced by PCR using S1 and S2 primers from the pNsbHLH2 vector. Ten μg of genomic DNA was digested by SspI and KpnI was separated by electrophoresis on 0.8 % agarose gels. After acid hydrolysis (0.25 M HCl), denaturation (0.5 M NaOH, 1.5 M NaCl), and neutralization (0.5 M TRIS–HCl pH 8.0, 1.5 M NaCl), DNA fragments were transferred to a Hybond-N^+^ nylon membrane (GE healthcare, UK) by capillary transfer in 10 × SSC (3 M NaCl, 0.3 M NaC_6_H_8_O_7_, and pH 7 with HCl) buffer overnight at room temperature. After UV-cross linking, the nylon membrane was pre-hybridized by the DIG-Easy-Hyb solution at 54 °C for 30 min. The DIG-labeled Sh*ble* probe was used for overnight hybridization at 54 °C in the DIG-Easy-Hyb solution. When using the DIG-labeled NsbHLH2 probe, hybridization was carried out at 52 °C. After 2 stringency washes (0.5 × SSC with 0.1 % SDS buffer at 65 °C for 15 min), detection was carried out using a block buffer and the anti-digoxigenin-alkaline phosphatase conjugate antibody (Roche, Germany), following the manufacture’s protocol.

### Determination of NsbHLH2 expression by quantitative real-time PCR (qRT-PCR) and Western blotting

*NsbHLH2* expression under different culture conditions was measured by qRT-PCR and Western blotting. cDNA was produced as described above, and qRT-PCR was carried out using the CFX96 Real-Time system (Bio-Rad, USA). The primers were designed to analyze expression levels of transgenic NsbHLH2 mRNA alone (qbH1 and qbH2) where qbH1 is located on the vector, and total NsbHLH2 mRNA (qbH3 and qbH4) (Additional file 5:Table S1). The housekeeping gene actin was used as a loading control and was amplified by qAT1 and qAT2 primers (Additional file 5: Table S1). The 20 μL reaction volume consisted of 2 μL of cDNA (for 20 ng of total RNA), 0.5 μL of 10 μM forward and reverse primers, 7 μL of DW, and 10 μL of Universal SYBR supermix (Bio-Rad, USA). The PCR consisted of the following steps: 95 °C for 2 min followed by 40 cycles of 95 °C for 10 s, 60 °C for 10 s, and 72 °C for 20 s, then 95 °C for 10 s, followed by a final melting step at 65-95 °C. Gene expression was determined by the 2^−∆∆Ct^ method, provided by the analysis program CFX Manager (Bio-Rad), and statistical significance was assessed by Students *t* test.

Western blotting was used to detect expression of FLAG-tagged NsbHLH2. For protein extraction, 1.5 × 10^8^ cells were suspended in 1.5 × modified Laemmli sample buffer (62.5 mM Tris–HCl, pH 7.6, 7 % sodium dodecyl sulfate [SDS], 25 % glycerol, 5 % β-mercaptoethanol, and 0.02 % bromophenol blue), and then incubated at 100 °C for 5 min [45]. After centrifugation at 13,000 rpm for 5 min, the supernatants were electrophoresed in SDS polyacrylamide gels (PAGE) with 4–15 % gradients [45]. The separated proteins were transferred to a polyvinylidene difluoride (PVDF) membrane by the Trans-Blot Turbo system (Bio-Rad). After blocking with 5 % skim milk and 0.1 % Tween 20 in phosphate-buffered saline (PBS), immunoblotting was carried out using a rabbit anti-FLAG-tag antibody (Cell Signaling Technology, USA) at a dilution of 1:1000 for 1 h. After washing, the membranes were incubated with horseradish peroxidase (HRP)-conjugated anti-rabbit secondary antibody (Cell Signaling Technology, USA) at a dilution of 1:1000 for 1 h. Protein bands were visualized using enhanced chemiluminescence (ECL) reagents and the ChemiDoc system (Bio-Rad).

### Fatty acid methyl ester (FAME) analysis

For lipid extraction, a chloroform–methanol mixture (2:1, v/v) was added to 10 mg of lyophilized cells and then vigorously mixed for 10 min. Heptadecanoic acid (C17:0, 0.5 mg) was added as an internal standard. For transesterification, 1 mL of methanol and 300 µL of sulfuric acid was added, and then incubated at 100 °C for 20 min. After cooling, 1 mL of deionized water was added and mixed. After centrifugation at 4000 rpm for 10 min, the organic phase (lower layer) was obtained and filtered using a 0.20-μm RC-membrane syringe filter (Sartorius Stedim Biotech, Germany). FAMEs were analyzed by a gas chromatograph (GC) (HP 6890, Agilent, USA) that had a flame ionization detector (FID) and an HP-INNOWax polyethylene glycol column (HP 19091 N-213, Agilent, USA). The oven temperature of the GC increased from 50 to 250 at 15 °C per min. FAME composition and content were determined based on a 37-component mix of FAME standards (F.A.M.E. MIX C8-C24, Supelco, USA).

## Additional files

10.1186/s13068-015-0386-9 Alignment of bHLH TFs from *N. gaditana* and *N salina* strains.
10.1186/s13068-015-0386-9 Western blotting of FLAG-tagged NsbHLH2.
10.1186/s13068-015-0386-9 Phenotype screening of NsbHLH2 transformants.
10.1186/s13068-015-0386-9 Growth curve of NsbHLH2 3–11 transformant and WT in the fed-batch cultivation.
10.1186/s13068-015-0386-9 Primers used in this study.
10.1186/s13068-015-0386-9 Supplementary methods.

## Abbreviations

TF: Transcription factor
bHLH: Basic helix-loop-helix
qRT-PCR: Quantitative real-time polymerase chain reaction
DCW: Dry cell weight
FAME: Fatty acid methyl ester
