# Supplementary material for: Effects of overexpression of a bHLH transcription factor on biomass and lipid production in Nannochloropsis salina
Source: Biotechnol Biofuels. 2015 Dec 1;8:200. doi: 10.1186/s13068-015-0386-9 (PMC4666162; doi:10.1186/s13068-015-0386-9)
Supplement: Supplementary file 1 — 10.1186/s13068-015-0386-9 Alignment of bHLH TFs from N. gaditana and N salina strains. [file 13068_2015_386_MOESM1_ESM.docx]

**(A)**


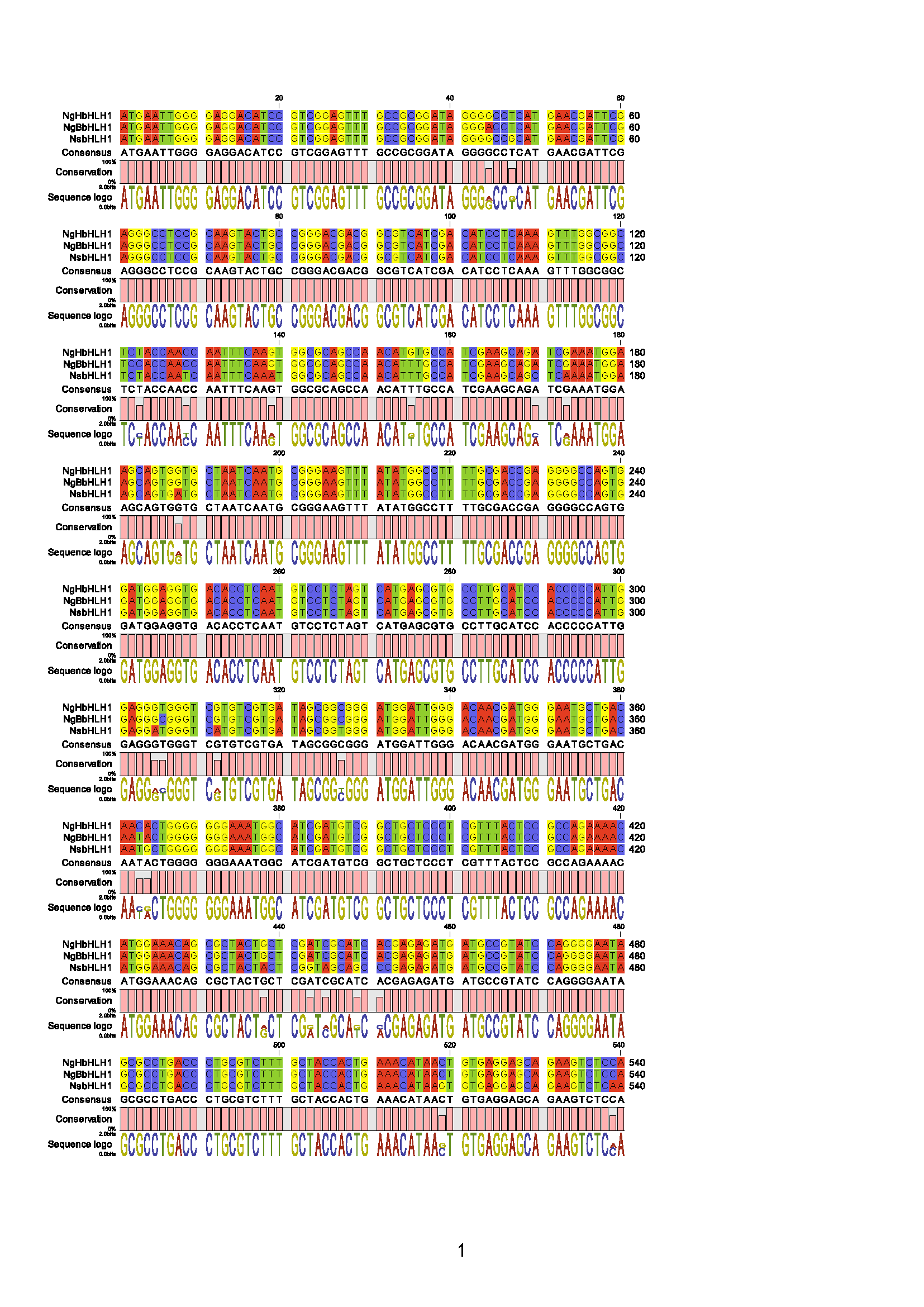

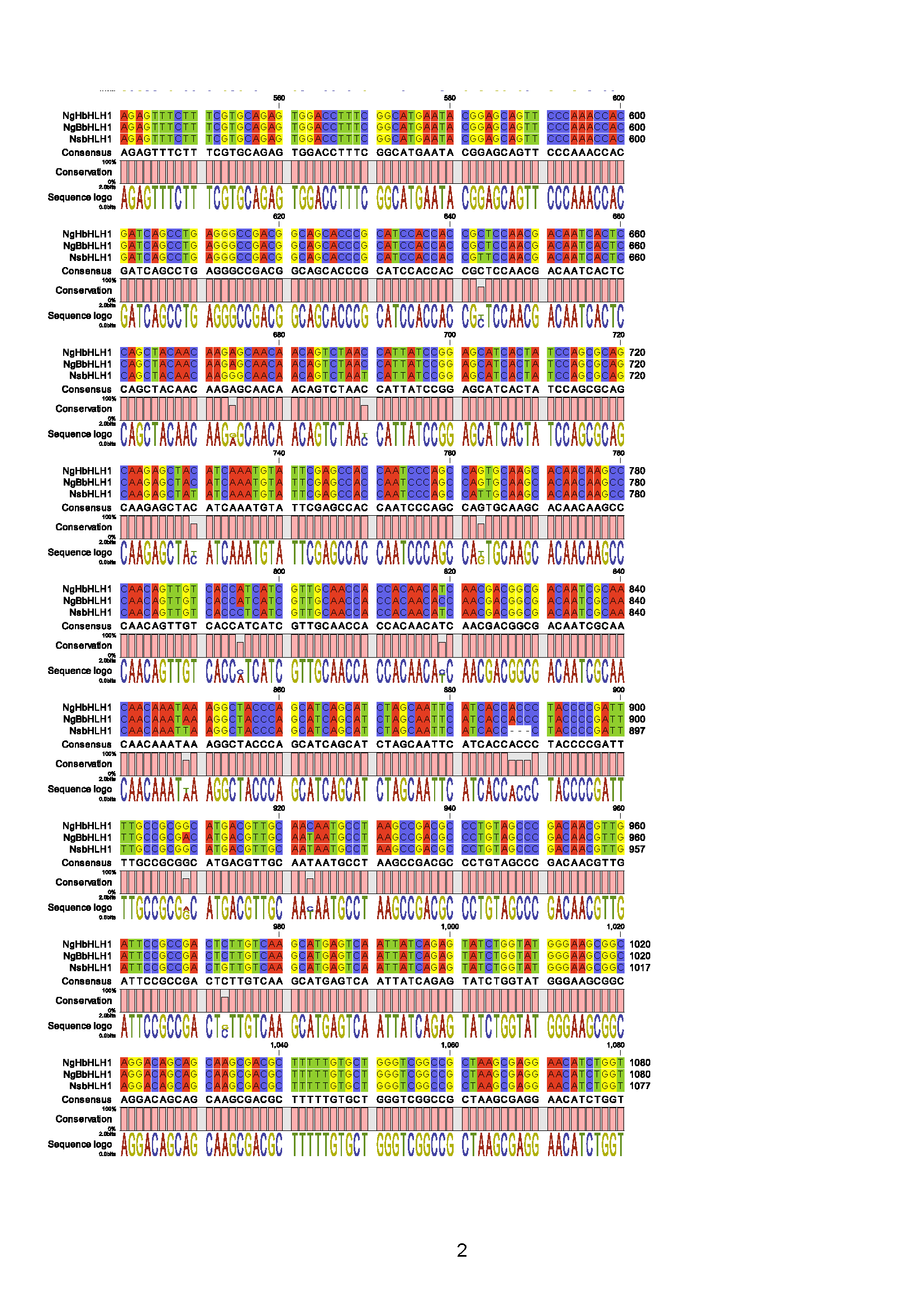

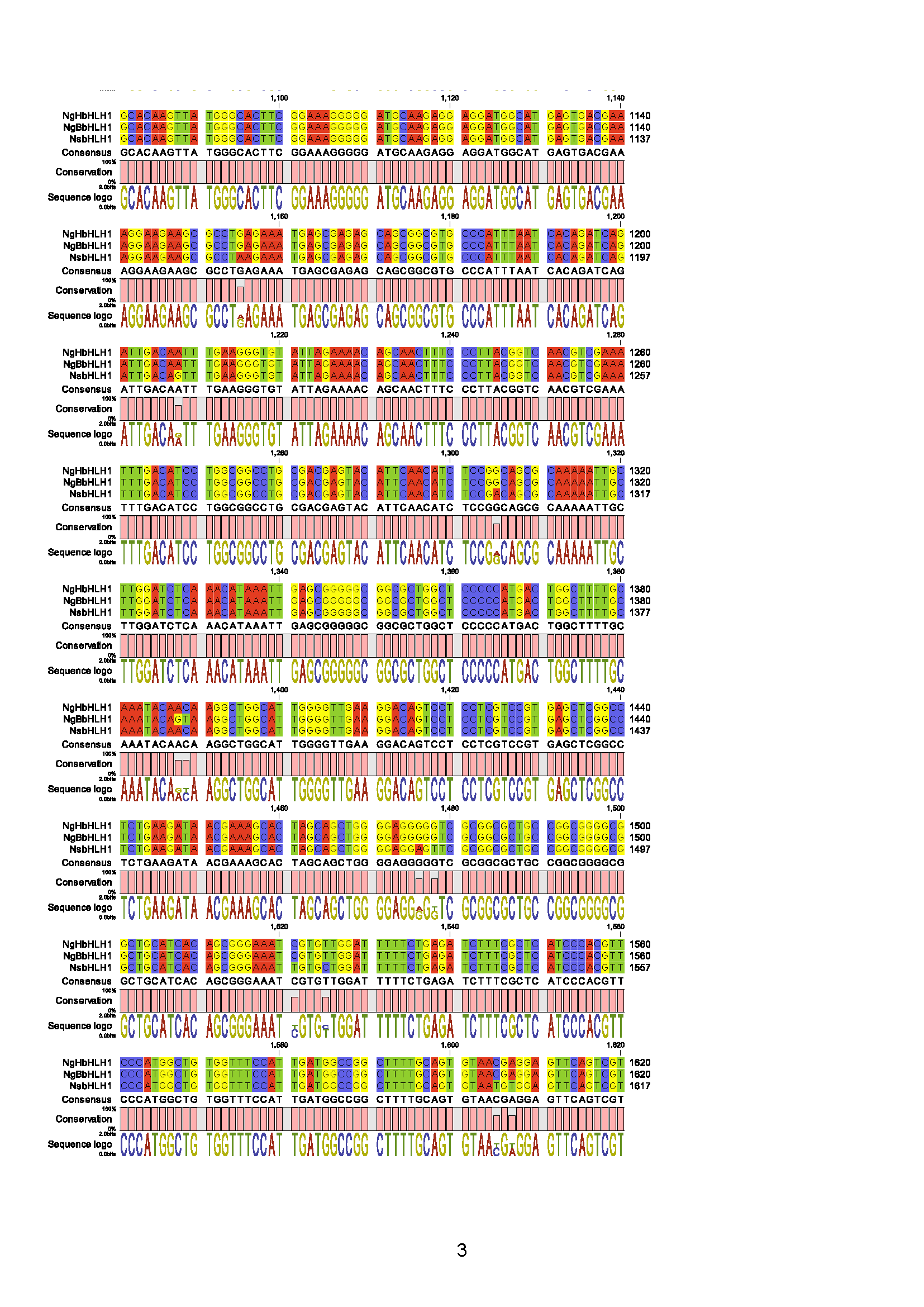

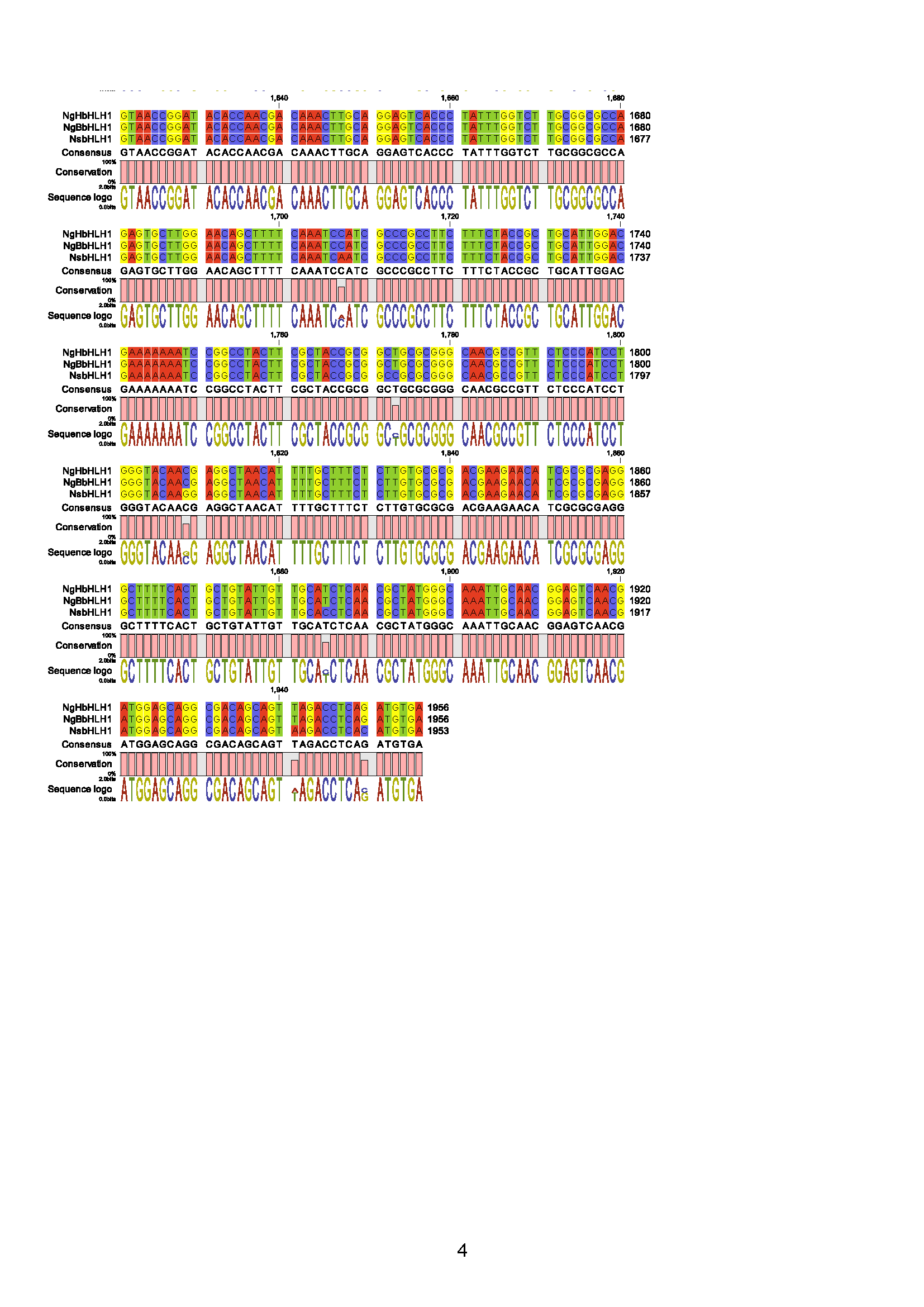


**(B)**

**
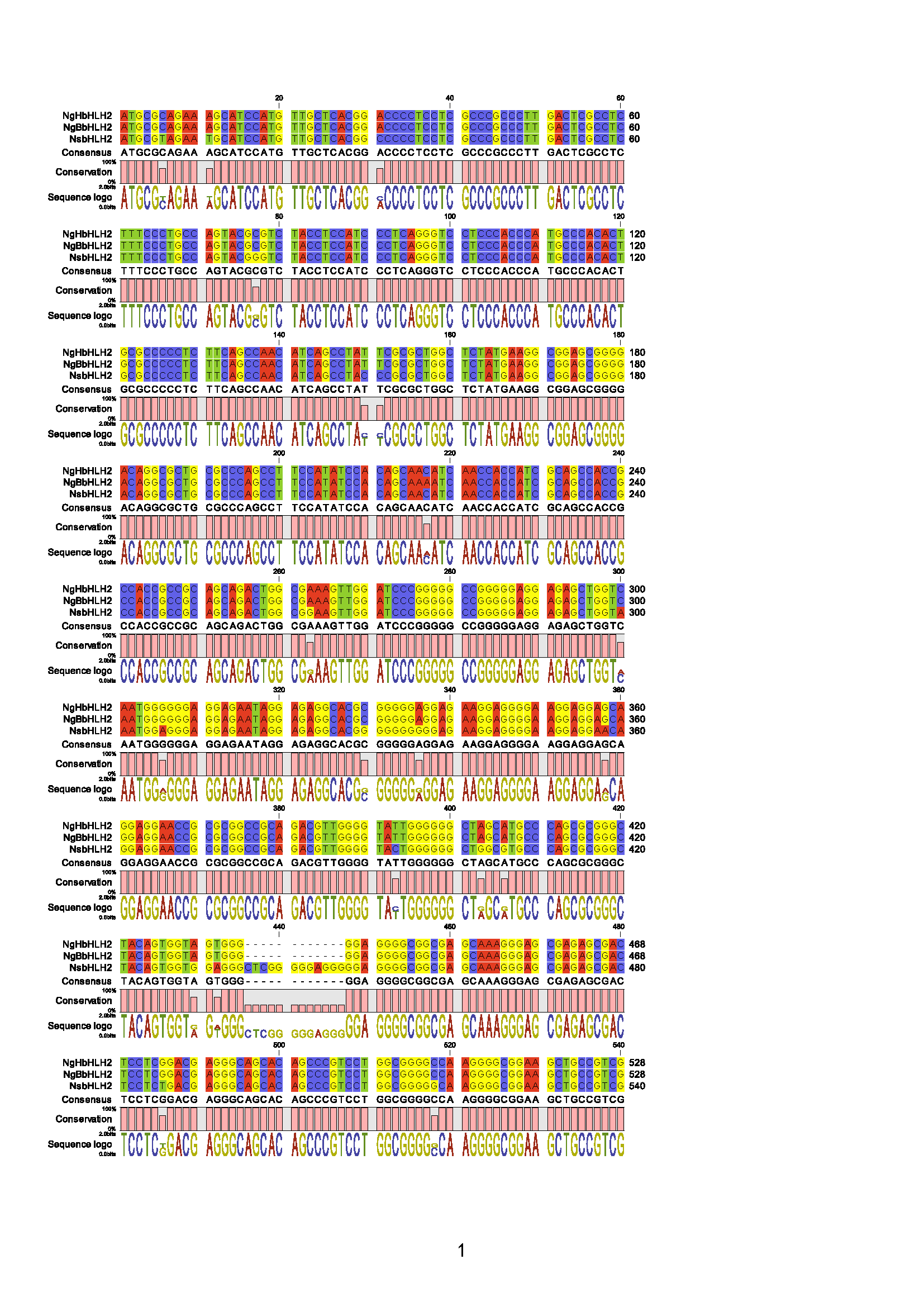

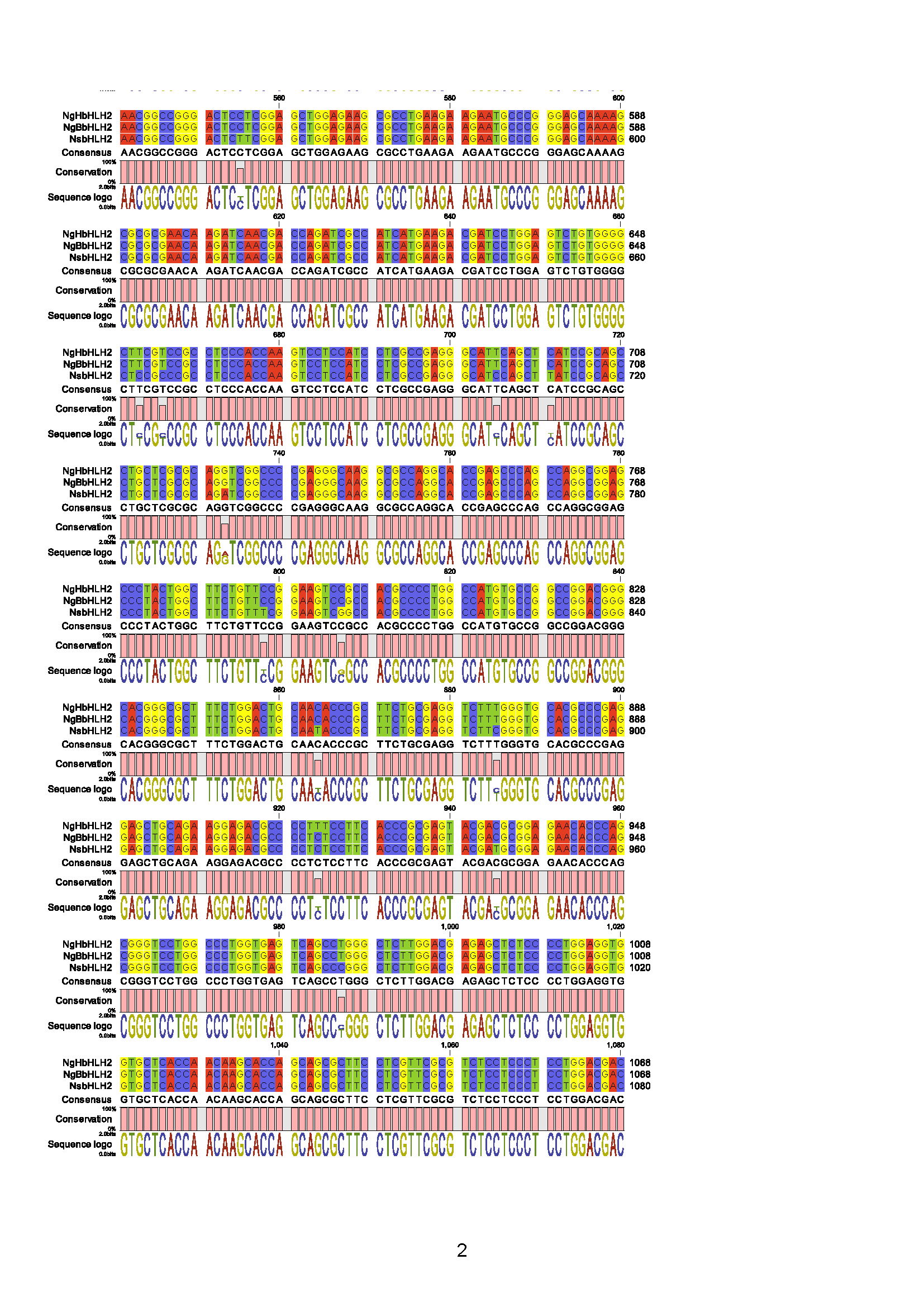

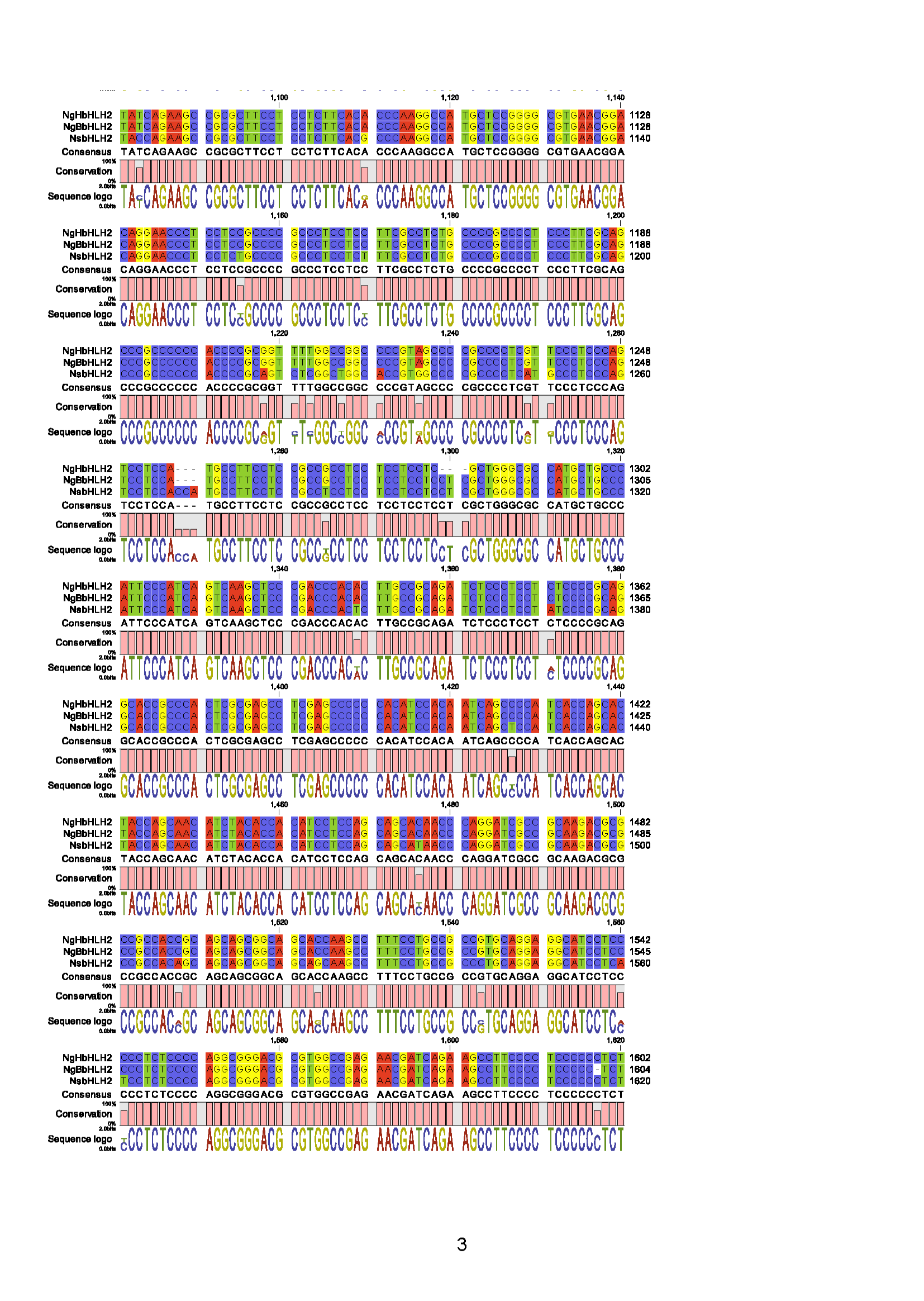

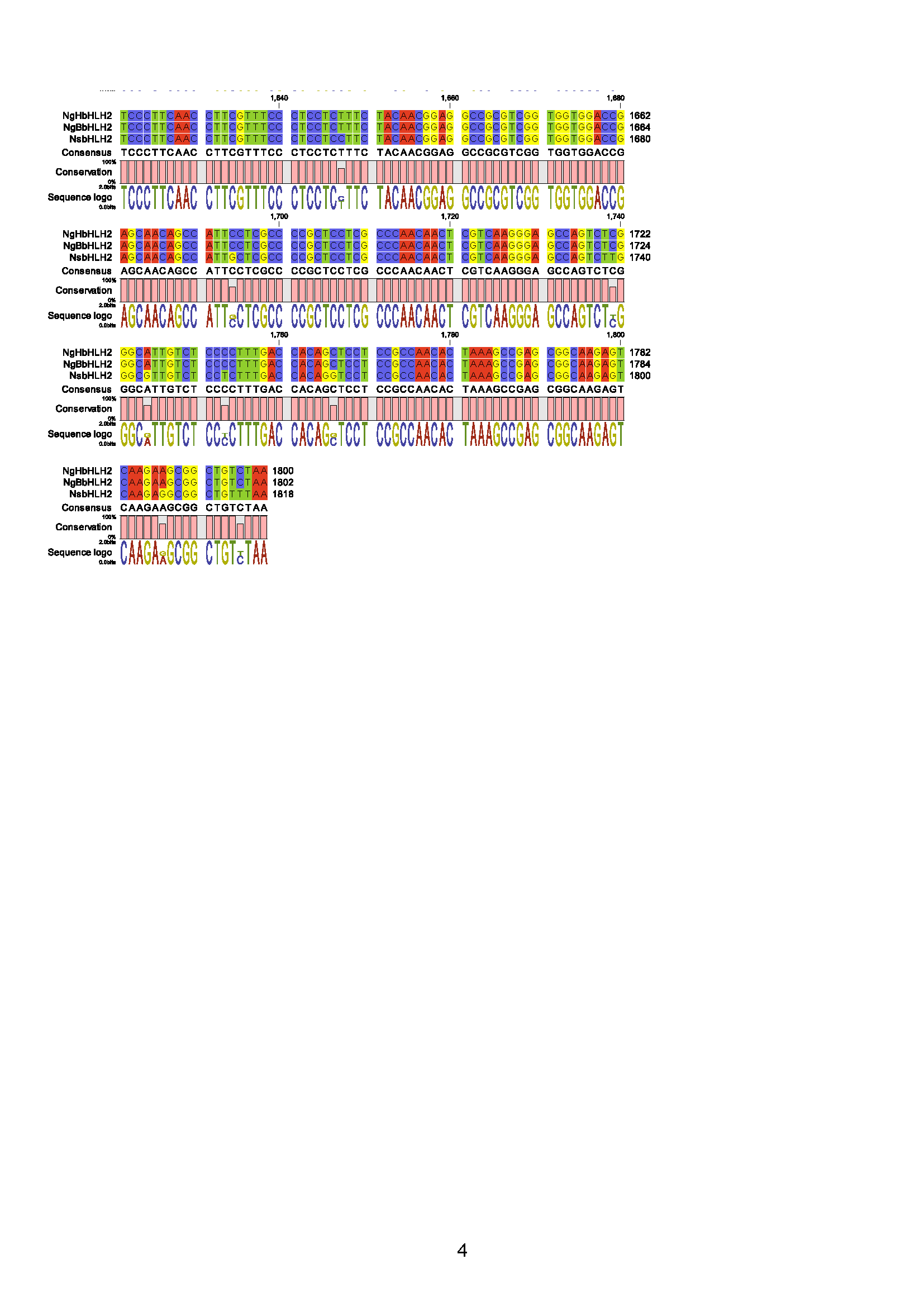
**

**Figure S1. Alignment of bHLH TFs from *N. gaditana* and *N salina* strains.** **(A)** bHLH1 homologs. **(B)** bHLH2 homologs.
