# Supplementary material for: Effects of overexpression of a bHLH transcription factor on biomass and lipid production in Nannochloropsis salina
Source: Biotechnol Biofuels. 2015 Dec 1;8:200. doi: 10.1186/s13068-015-0386-9 (PMC4666162; doi:10.1186/s13068-015-0386-9)
Supplement: Supplementary file 2 — 10.1186/s13068-015-0386-9 Western blotting of FLAG-tagged NsbHLH2. [file 13068_2015_386_MOESM2_ESM.docx]

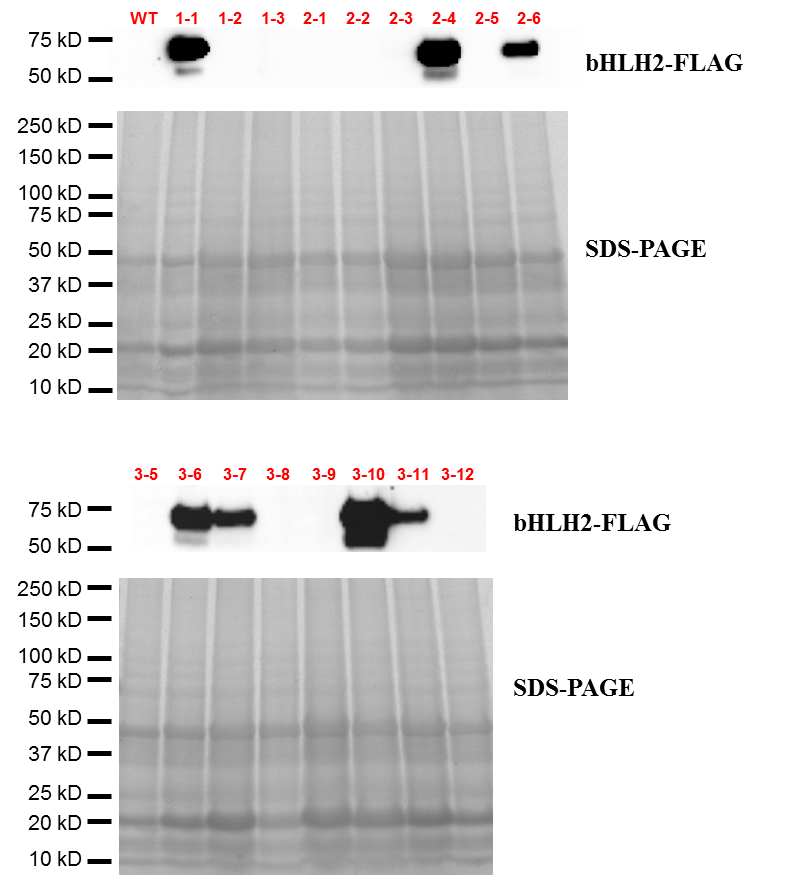


**Figure S2.** **Western blotting of FLAG-tagged NsbHLH2.** Stain-free SDS-PAGE gel image using ChemiDoc (Bio-Rad) showed similar loading of protein samples. The expected size of FLAG-tagged NsbHLH2 was 65 kD.
