# Supplementary material for: Effects of overexpression of a bHLH transcription factor on biomass and lipid production in Nannochloropsis salina
Source: Biotechnol Biofuels. 2015 Dec 1;8:200. doi: 10.1186/s13068-015-0386-9 (PMC4666162; doi:10.1186/s13068-015-0386-9)
Supplement: Supplementary file 3 — 10.1186/s13068-015-0386-9 Phenotype screening of NsbHLH2 transformants. [file 13068_2015_386_MOESM3_ESM.docx]

**
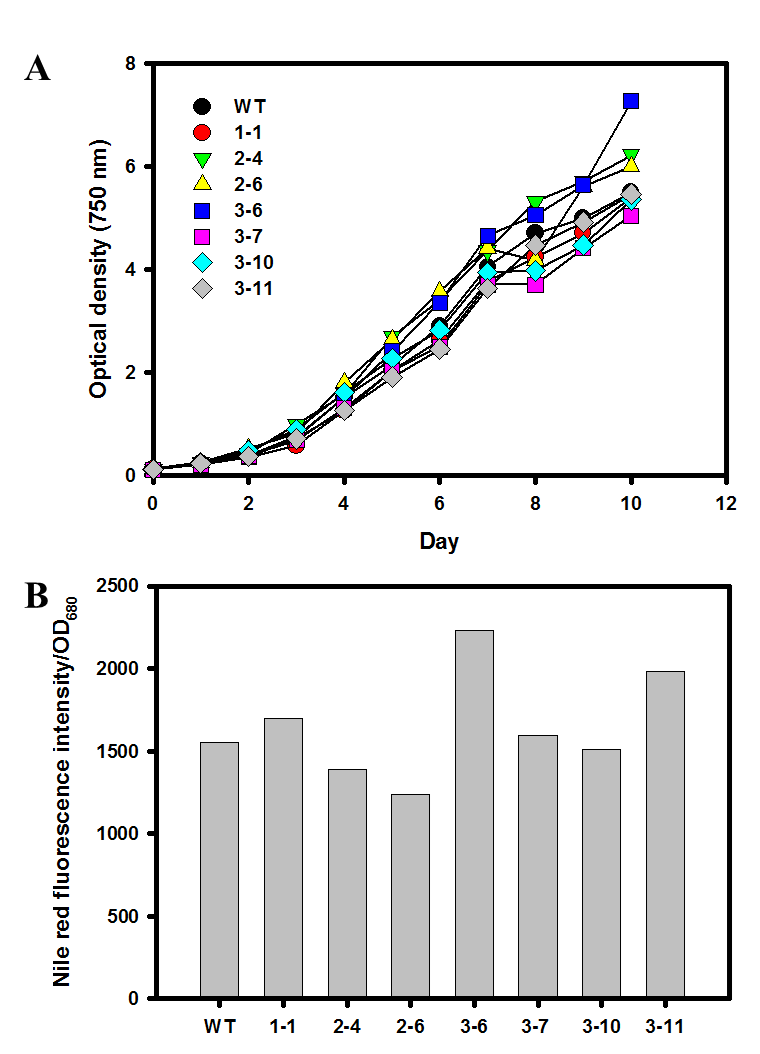
**

**Figure S3.** **Phenotype screening of NsbHLH2 transformants.** **(A)** Growth curve by optical density at 750 nm. **(B)** Relative neutral lipid contents by Nile red fluorescence intensity.
