# Supplementary material for: Effects of overexpression of a bHLH transcription factor on biomass and lipid production in Nannochloropsis salina
Source: Biotechnol Biofuels. 2015 Dec 1;8:200. doi: 10.1186/s13068-015-0386-9 (PMC4666162; doi:10.1186/s13068-015-0386-9)
Supplement: Supplementary file 4 — 10.1186/s13068-015-0386-9 Growth curve of NsbHLH2 3–11 transformant and WT in the fed-batch cultivation. [file 13068_2015_386_MOESM4_ESM.docx]

**Figure S4.** **Growth curve of NsbHLH2 3-11 transformant and WT in the fed-batch cultivation.** At day 6 and 10, 1.14 mL of solution containing 85.5 mg NaNO_3_ and 1 mL containing 6 mg NaH_2_PO_4_∙2H_2_O were added to supply nitrogen and phosphorus sources. Cells were cultivated at 25°C, 120 rpm, 120 µmol photons/m^2^/s of fluorescent light, and 0.5 vvm of 2% CO_2_. Data points represent means and standard errors of triplicate samples (n = 3).
