# Supplementary material for: Effects of overexpression of a bHLH transcription factor on biomass and lipid production in Nannochloropsis salina
Source: Biotechnol Biofuels. 2015 Dec 1;8:200. doi: 10.1186/s13068-015-0386-9 (PMC4666162; doi:10.1186/s13068-015-0386-9)
Supplement: Supplementary file 5 — 10.1186/s13068-015-0386-9 Primers used in this study. [file 13068_2015_386_MOESM5_ESM.docx]

**Table S1.** **Primers used in this study.**

|  | Primer | Sequence 5'-3' | Purpose |
| --- | --- | --- | --- |
|  | bH1 | TCAAGAAGCTGTCTTTTTGTGAAGCATGCGTAGAATGCATCCATGTTGCTC | Amplifying bHLH2 cDNA for Gibson assembly |
|  | bH2 | AAAAGTGGTCGGACAAAAGGAGTTTCACTTGTCGTCGTCGTCCTTGTAGTC |  |
|  | bH3 | GACTACAAGGACGACGACGACAAGTGAAACTCCTTTTGTCCGACCACTTTTACAATC | Amplifying pNsbHLH2 backbone for Gibson assembly |
|  | bH4 | AGCAACATGGATGCATTCTACGCATGCTTCACAAAAAGACAGCTTCTTGATATTGACTTT |  |
|  | S1 | AAGTTGACCAGTGCCGTTCCGGTG | Obtaining Sh*ble* probe for Southern blot and colony PCR |
|  | S2 | CTCGGTCACGAAGTGCACGCAGTT |  |
|  | SR6 | GTCAGAGGTGAAATTCTTGG | 18s rDNA |
|  | SR9 | AACTAAGAACGGCATGCAC |  |
|  | qbH1 | GAACAAGATCAACGACCAGAT | qRT-PCR for transgenic bHLH2 mRNA |
|  | qbH2 | CGAGGATGGAGGACTTGG |  |
|  | qbH3 | AGATAAAGGTCAATATCAAGAAGC | qRT-PCR for transgenic and endogenous bHLH2 mRNA |
|  | qbH4 | GAAAGAGGCGAGTCAAGG |  |
|  | qAT1 | GTGTTTCCCTCCATCGTG | qRT-PCR for Actin mRAN |
|  | qAT2 | CCAGTTCGTCACAATACCG |  |
