# Supplementary material for: Effects of overexpression of a bHLH transcription factor on biomass and lipid production in Nannochloropsis salina
Source: Biotechnol Biofuels. 2015 Dec 1;8:200. doi: 10.1186/s13068-015-0386-9 (PMC4666162; doi:10.1186/s13068-015-0386-9)
Supplement: Supplementary file 6 — 10.1186/s13068-015-0386-9 Supplementary methods. [file 13068_2015_386_MOESM6_ESM.docx]

**Text S1. Supplementary methods**

1. Fed-batch cultivation

In order to conduct lab scale fed-batch cultivation, we used modified F2N medium [1]. Cells were cultivated in 250 mL Erlenmeyer baffled flasks with 200 mL working volumes at 25°C with agitation (120 rpm) under the fluorescent light (120 µmol photons/m^2^/s). Air with 2% CO_2_ was supplied to the broth culture at 0.5 vvm (volume gas per volume medium per minute). At days 6 and 10, 1.14 mL containing of 85.5 mg NaNO_3_ and 1 mL containing 6 mg were added to supply nitrogen and phosphorus sources.

1. Nile red staining and quantification of neutral lipid contents

Neutral lipid contents were determined by Nile red assay [2]. At the final day of cultivation, samples were diluted to make the same OD680 of 0.1. Cells were stained with Nile red (5 µg/mL) in black 96 well plates. The cells were incubated at 37ºC for 30 min, and fluorescence intensity was analyzed by SpectraMax M2 spectrophotometer (Molecular Devices, USA). A wavelength of 530 nm was used for excitation and 575 nm for emission.

**Reference**

1. Kang, N.K., B. Lee, S.E. Shin, S. Jeon, M.S. Park, and J.W. Yang: **Use of conditioned medium for efficient transformation and cost-effective cultivation of *Nannochloropsis salina*.** *Bioresour Technol* 2015, **181:**231-237.

2. Chen, W., C. Zhang, L. Song, M. Sommerfeld, and Q. Hu: **A high throughput Nile red method for quantitative measurement of neutral lipids in microalgae.** *J Microbiol Methods* 2009, **77:**41-47.
